# Plasma and Salivary Non-Urate Total Antioxidant Capacity Does Not Depend on Dietary Vitamin C, E, or β-Carotene Intake in Older Subjects

**DOI:** 10.3390/molecules23040983

**Published:** 2018-04-23

**Authors:** Anna Gawron-Skarbek, Agnieszka Guligowska, Anna Prymont-Przymińska, Dariusz Nowak, Tomasz Kostka

**Affiliations:** 1 Department of Hygiene and Health Promotion, Medical University of Lodz, Hallera St. 1, 90-647 Łódź, Poland; 2 Department of Geriatrics, Medical University of Lodz, Pieniny St. 30, 90-993 Łódź, Poland; agnieszka.guligowska@umed.lodz.pl (A.G.); tomasz.kostka@umed.lodz.pl (T.K.); 3 Department of General Physiology, Medical University of Lodz, Mazowiecka St. 6/8, 92-215 Łódź, Poland; anna.przyminska@umed.lodz.pl; 4 Department of Clinical Physiology, Medical University of Lodz, Mazowiecka St. 6/8, 92-215 Łódź, Poland; dariusz.nowak@umed.lodz.pl

**Keywords:** non-urate total antioxidant capacity, plasma, saliva, nutrition, habitual diet, vitamin C intake, vitamin E intake, β-carotene, DPPH, FRAP

## Abstract

The native Total Antioxidant Capacity (TAC) of plasma and saliva is generally determined by uric acid (UA). Several studies have assessed the impact of habitual dietary antioxidative vitamin intake on TAC, but it remains unknown whether it influences Non-Urate Total Antioxidant Capacity (Nu-TAC), i.e., TAC after enzymatic UA elimination. The purpose of this study was to assess whether the intake of antioxidative vitamins C, E, and β-carotene, provided with usual daily food rations, affects plasma and salivary Nu-TAC. The study involved 56 older subjects (aged 66.9 ± 4.3 years), divided into two age- and sex-matched groups: group 1 (*n* = 28), with lower combined vitamin C, E, and β-carotene intake, and group 2 (*n* = 28), with higher intake. A 24 h dietary recall was obtained from each individual. Nu-TAC was assessed simultaneously with two methods in plasma (Ferric Reducing Ability of Plasma—Nu-FRAP, 2.2-diphenyl-1-picryl-hydrazyl—Nu-DPPH) and in saliva (Nu-FRAS and Nu-DPPHS test). No differences were found in the Nu-TAC parameters between the groups, either in plasma (Nu-FRAP, Nu-DPPH) or in saliva (Nu-FRAS, Nu-DPPHS) (*p* > 0.05). No plasma or salivary Nu-TAC indices correlated with dietary vitamin C, E, or β-carotene intake or with other nutrients. Habitual, not extra-supplemented dietary intake does not significantly affect plasma or salivary Nu-TAC.

## 1. Introduction

The balance between pro-oxidants and antioxidants in living organisms prevents oxidative stress, which is regarded as a cause of numerous chronic diseases, including cardiovascular heart disease, diabetes mellitus type 2, and periodontitis [1,2]. Reactive oxygen and nitrogen species (ROS/RNS) produced under oxidative stress are known to damage all cellular biomolecules (lipids, sugars, proteins, and polynucleotides), [3] but a cellular antioxidant defense system is known to counteract uncontrolled ROS increase. The system is formed by non-enzymatic molecules (uric acid, vitamins C, E, carotenoids, polyphenols, glutathione) and enzymatic scavengers of ROS (superoxide dismutase, catalase, glutathione peroxidase) [4]. Although there are many external (diet, physical activity, cigarette smoking, environmental pollutants, radiations) and internal (biochemical disorders) factors that might enhance or limit the action of the endogenous antioxidant system, it is not clear whether an increased body fluid antioxidant capacity is a desirable condition if it reflects a response to increased oxidative stress. The results of studies assessing the impact of dietary supplementation with various antioxidant compounds in Daily Food Rations (DFR) mostly indicate temporary augmentation of the antioxidant status [5]. Such dietary interventions are usually associated with higher consumption of fruits, vegetables, and plant oils as main food sources of antioxidative compounds [6,7]. As vitamin C, E, and β-carotene are known to be representative dietary antioxidants, their high content in the DFR is expected to enhance the antioxidant potential in body fluids, cells, and tissues. However, limited information is available on whether the differences in antioxidant capacity observed between different body fluids reflect a habitual dietary intake of antioxidants [8,9]. Subjects following a naturally antioxidant-rich diet, without any special regimens, might experience different biological effects than those being supplemented by multivitamins and minerals [10]. It is thought that the protective effects of fruit and vegetable consumption result from the presence of low-molecular antioxidants, such as α-tocopherol, ascorbic acid, or β-carotene, as well as non-vitamin antioxidants, such as polyphenols and anthocyanins, or from the synergy of several different antioxidant compounds [7]. Other reports indicate that vitamin C may exert a pro-oxidant effect, especially in people receiving doses exceeding the daily recommended dietary allowance [11].

A variety of methods are used to assess the antioxidant defense system, providing a range of results which are at times inconsistent. A holistic assessment of Total Antioxidant Capacity (TAC) may be a better approach than determining the capacities of individual antioxidants. There are methods that measure only the non-enzymatic antioxidant capacity of deproteinized native plasma or saliva specimens, such as the 2.2-diphenyl-1-picryl-hydrazyl test (DPPH/DPPHS), and others suited to samples with a lower sensitivity to protein antioxidants, such as the Ferric Reducing Ability of Plasma (FRAP) or of Saliva (FRAS). Saliva may represent an alternative noninvasive means for evaluating the impact of dietary antioxidant intake on the plasma antioxidant defense system. Nonetheless, in view of the predominant contribution of uric acid (UA) to plasma TAC (about 64–76%) as well as of salivary uric acid (SUA) to salivary TAC (about 72–75%) [12,13], the Non-urate TAC (Nu-TAC) of plasma (Nu-FRAP or Nu-DPPH) or saliva (Nu-FRAS or Nu-DPPHS), assessed after UA/SUA elimination using uricase, seems to be a more sensitive marker of the antioxidant status than native TAC. Individual differences in UA concentration may also explain many of the differences in antioxidant capacity [14] associated with the applied method, its limitations, or the type of study. These complications suggest that a “battery” of measurements (including different biological fluids and assays of native and non-urate TAC with different methods) would be more effective at assessing the antioxidant barrier level and oxidative stress in biological systems than any single measurement of the antioxidant status [15].

The aim of this study was to assess the impact of nutrients, mostly the antioxidative vitamins C, E, and β-carotene, obtained from DFR on plasma and salivary Nu-TAC parameters in older adults.

## 2. Results

### 2.1. Baseline Group Characteristics

Detailed demographic, anthropometric, and laboratory characteristics of the studied groups are shown in Table 1. The two subgroups did not differ with regard to age. Over 2/5 of the group were diagnosed with obesity, and further 1/3 of the group with overweight. Visceral obesity was found in almost 3/4 of the group. Group 1 and 2 were similar with regard to the anthropometric and lipid profile parameters, except for low-density lipoprotein cholesterol (LDL–Ch) and triglycerides (TG): group 2 had a lower TG but a higher LDL–Ch concentration (*p* < 0.05).

### 2.2. Total and Non-Urate Antioxidant Indices

Table 2 presents the mean values of non-urate and total plasma and salivary antioxidant indices assessed with the FRAP/FRAS and DPPH/DPPHS tests, respectively. No differences were found for Nu-TAC parameters between the groups, either in plasma or in saliva (for all *p* > 0.05).

FRAS (r = 2.4; *p* < 0.05) and DPPH (r = 2.3, *p* < 0.05) were decreased in group 2, but no other group differences were found for total salivary or for total plasma antioxidant parameters.

None of the tested parameters, i.e., UA (4.7 ± 1.0 vs. 4.3 ± 1.3 mg dL^−1^), SUA (9.5 ± 2.9 vs. 7.6 ± 3.7 mg dL^−1^), salivary C-Reactive Protein (CRP) (2.21 ± 2.08 vs. 2.19 ± 1.87 ng mL^−1^), ∆ FRAP (65.2 ± 7.1 vs. 64.4 ± 7.2%), ∆ DPPH (78.1 ± 5.6 vs. 74.0 ± 11.4%), ∆ FRAS (75.6 ± 10.3 vs. 70.8 ± 11.8%), and ∆ DPPHS (78.8 ± 11.3 vs. 72.8 ± 13.5%) showed any difference between the groups (for all *p* > 0.05, data not shown in the Table).

### 2.3. Nutritional Characteristics

Several similarities in nutritional compound intake were found between the groups: the absolute values of the energy obtained from particular macronutrient intake (19.6% from proteins, 28.5% from fat, and 51.7% from carbohydrates), total fat, saturated and monounsaturated fatty acids, cholesterol, animal protein, vitamin B_1_, B_12_, niacin, sodium, calcium, and manganese were similar (data not shown in the figure).

**Figure 1:**
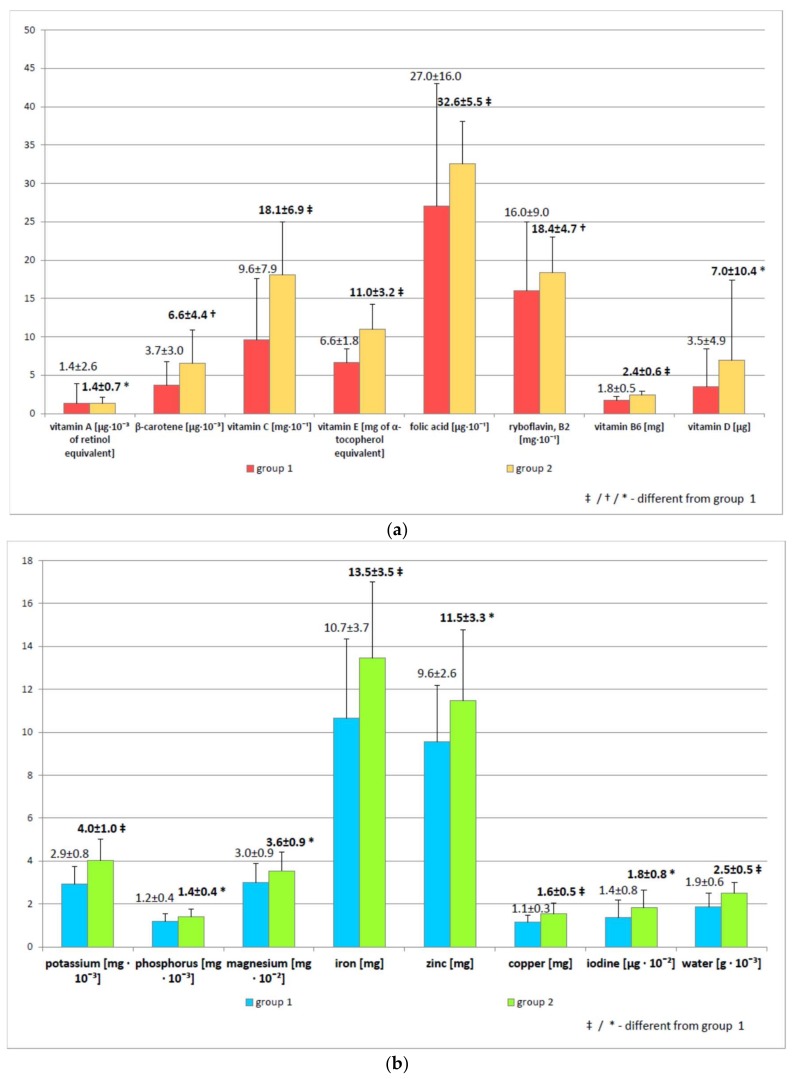

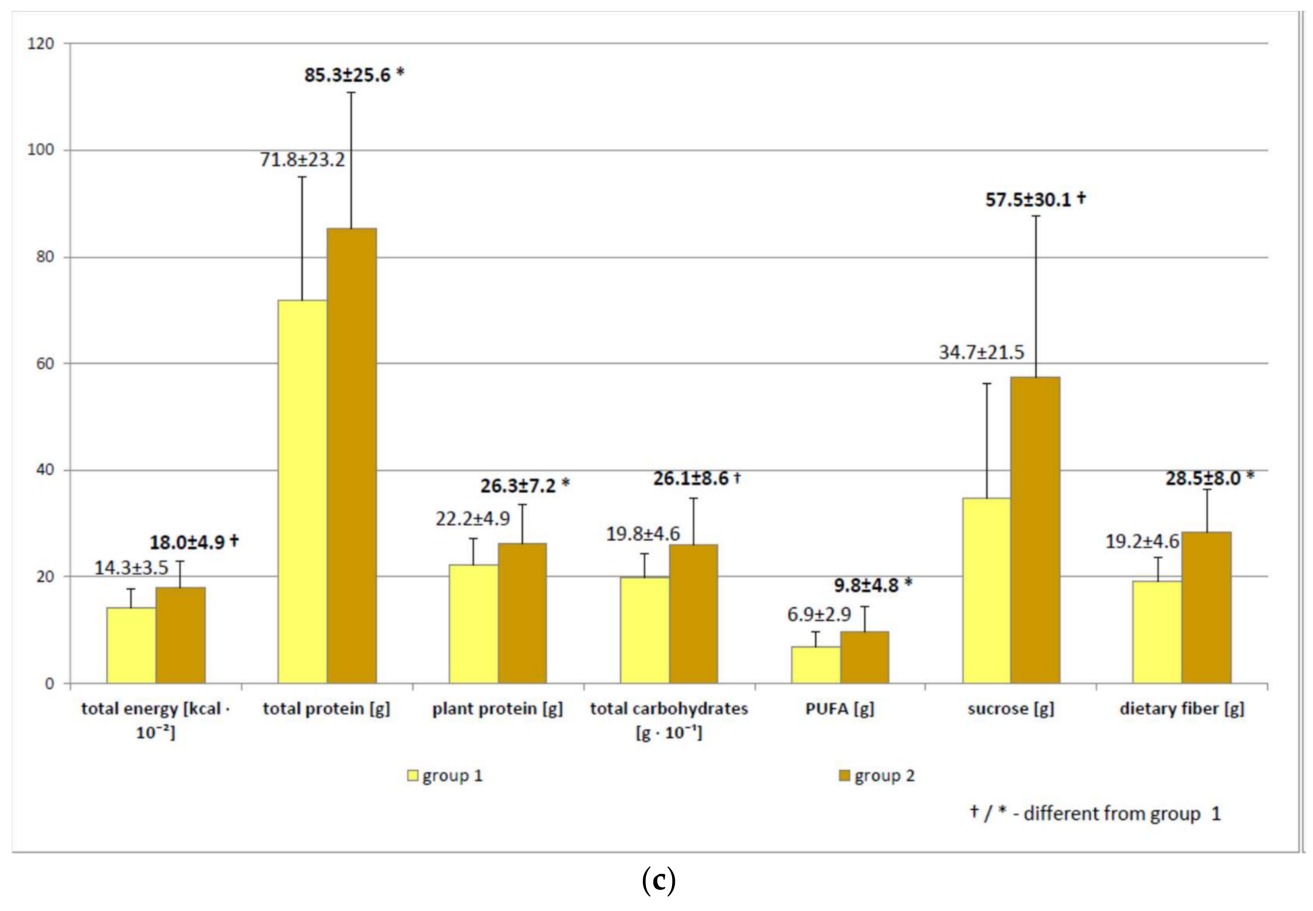
Comparison of daily intake of selected nutrients between the study groups: (**a**) vitamins; (**b**) minerals and water; (**c**) energy and other nutrients. The data are presented as mean ± SD. PUFA—Polyunsaturated Fatty Acids; *—*p* < 0.05, †—*p* < 0.01, ‡—*p* < 0.001 as compared to group 1.

As expected, vitamin C (96.3 ± 79.4 mg vs. 180.7 ± 68.8 mg, r = −4.8, *p* < 0.001), vitamin E (6.6 ± 1.8 mg vs. 11.0 ± 3.2 mg, r = −5.2, *p* < 0.001), and β-carotene intakes (3742.1 ± 3033.3 µg vs. 6551.7 ± 4390.7 µg, r = −3.0, *p* < 0.01) were significantly higher in group 2 (Figure 1a). Total energy (*p* < 0.01), total protein (*p* < 0.05), plant protein (*p* < 0.05), total carbohydrates (*p* < 0.01), sucrose (*p* < 0.01), dietary fiber (*p* < 0.05), and polyunsaturated fatty acids (*p* < 0.05) were significantly higher in group 2 (Figure 1b), as was the intake of water (*p* < 0.001) and some minerals (potassium (*p* < 0.001), phosphorus (*p* < 0.05), magnesium (*p* < 0.05), iron (*p* < 0.001), zinc (*p* < 0.05), copper (*p* < 0.001), iodine (*p* < 0.05)) (Figure 1c), vitamins (B_2_ (*p* < 0.01), B_6_ (*p* < 0.001), A (*p* < 0.05), and D (*p* < 0.05), and folic acid (*p* < 0.001)) (Figure 1a).

### 2.4. Correlations for Non-Urate and Total Antioxidant Indices in the Study Group (n = 56)

Age was not correlated with any native or non-urate antioxidant indices, either in plasma or in saliva. Individuals with higher Body Mass Index (BMI) had increased UA (r = 0.30) and salivary CRP (r = 0.36), while only the plasma Nu-TAC parameters were decreased (Nu-FRAP—r = −0.37 and Nu-DPPH—r = −0.41; *p* < 0.01). Participants with higher TG had increased FRAP (r = 0.38), DPPH (r = 0.27), and UA (r = 0.27), and those with higher High-Density Lipoprotein Cholesterol (HDL–Ch) had decreased FRAP (r = −0.35). Subjects with visceral obesity were characterized by higher FRAP (r = 0.29), UA (r = 0.46), and SUA (r = 0.29), and lower plasma Nu-TAC indices (Nu-FRAP—r = −0.26 and Nu-DPPH—r = −0.27; *p* < 0.05).

No plasma or salivary Nu-TAC indices correlated with dietary vitamin C, E, or β-carotene intake or with other nutrients (*p* > 0.05). Higher dietary fiber intake corresponded to lower FRAS (r = −0.34) and lower FRAP (r = −0.28), but higher vitamin C, zinc, and sodium intake corresponded to lower FRAS alone (r = −0.28, r = −0.33 and r = −0.28, respectively). No other correlations were observed between vitamin C, E, or β-carotene intake and antioxidant indices or salivary CRP. No native antioxidant parameter, either in saliva or in plasma, was related to salivary CRP (*p* > 0.05), but higher salivary CRP was related to lower Nu-FRAP (r = −0.31).

No correlations were found between plasma (Nu-FRAP, Nu-DPPH) and salivary (Nu-FRAS, Nu-DPPHS) non-urate antioxidant indices, but Nu-FRAP positively corresponded to Nu-DPPH (r = 0.46), as did Nu-FRAS to Nu-DPPHS (r = 0.27). Conversely, all total plasma antioxidant parameters (FRAP, DPPH, UA) correlated positively with their saliva analogues (FRAS, DPPHS, SUA) (*p* < 0.05) (data not shown in the Table).

## 3. Discussion

A previous methodological study [12] compared plasma native TAC and Nu-TAC to salivary analogues. This is the first study to assess the impact of nutrients, mostly the antioxidative vitamins C, E, and β-carotene, on plasma (Nu-FRAP and Nu-DPPH) and salivary Nu-TAC (Nu-FRAS and Nu-DPPHS test) parameters in older adults, and one of the very few studies that simultaneously assesses native TAC by two different established methods in plasma (FRAP and DPPH) and in saliva (FRAS and DPPHS test) in a group of relatively healthy older adults. The present study is an extension of our previously published results of a cross-sectional study which found that dietary vitamin C, E, and β-carotene obtained from DFR did not significantly affect native plasma or salivary TAC, UA/SUA, and salivary CRP [16]. It examines two subgroups, in which the native and Nu-TAC indices of plasma and saliva were assayed jointly to evaluate the possible effect of habitual, not interventional, dietary antioxidative vitamin intake on UA-independent antioxidant capacity, by eliminating UA from the sample. Our current findings indicate that the level of typical, non-supplemented consumption of antioxidative vitamins with DFR did not affect the Nu-TAC or TAC of plasma or saliva, although an adverse effect on FRAS was noted in individuals with a higher level of dietary vitamin C intake. The nutritional status of group 2 was significantly superior to that of group 1, but generally the non-urate and native antioxidant status of both groups, besides FRAS and DPPH indexes, was comparable. Also, the two groups demonstrated similar UA, SUA, and salivary CRP concentrations, as well as plasma and salivary Δ TAC, regardless of any difference in combined vitamin C, E, and β-carotene intake.

Numerous interventional studies have assessed the potential impact of various nutritional compounds added to food [17,18] or beverages [19] in DFR on the antioxidant potential, or have examined the effect of individual antioxidants (UA, vitamin C, antioxidative enzymes) in plasma or serum, although few have used saliva. However, few studies have evaluated the influence of habitual dietary intake on the antioxidant status, especially its salivary non-urate fraction, which prevents a reliable comparison with the obtained data. The intake of dietary vitamins with fruits, vegetables, or plant oils is known to have a positive effect on health [20]; however, relatively little is known of their impact on the antioxidant potential indices (native and non-urate as well).

Nonetheless, studies on saliva samples, which require noninvasive techniques for determining the antioxidant parameters, are an appealing new research direction [21]. In addition, studies on non-urate plasma samples also offer promise as a means of TAC assessment; however, these studies have provided varying results. In an interventional study by Prymont-Przymińska et al. [22], it was found that the consumption of 500 g of strawberries (as a source of anthocyanins and ellagitanins, which have antioxidant properties) daily for nine days in a group of healthy adults was associated with an increase in fasting plasma Nu-DPPH activity but had no effect with regard to native plasma antioxidant activity. An intervention based on strawberry consumption was associated with an increase in postprandial, not fasting, Nu-FRAP and Nu-DPPH values (*p* < 0.05); however, in native plasma, only higher postprandial values were observed in both tests [23]. This is in contrast to the results of our non-interventional study, where both the fasting plasma non-urate antioxidant indices (Nu-FRAP and Nu-DPPH) and the salivary non-urate antioxidant indices (Nu-FRAS and Nu-DPPHS) were not influenced by the level of antioxidative vitamins intake.

An in vitro study by Rabovsky et al. found that the addition of 1200 mg vitamin C caused an amplified antioxidant protective effect against free radicals in plasma after enzymatic UA removal (Nu-FRAP) compared to native plasma samples (FRAP) [24]. These findings indicate that, once UA was removed, the antioxidant protection of other antioxidants present in the plasma (such as vitamins, phytochemicals, carotenoids, etc.) could be more demonstrable after supplementation with a high dose of vitamin C. Elevations in plasma UA concentration are found after intake of purine-rich red meat, fructose, or alcohol, as a result of renal disease, and in cases of tissue destruction, such as those following intensive exercise, and this can elevate the native TAC of the biological fluids and possibly mask changes in other antioxidants in plasma [25,26].

In contrast, increased levels of ascorbic acid in plasma were associated with decreased UA, as vitamin C has an uricosuric effect [27]. Therefore, in certain diseases, and after ingestion of certain food or supplements, the plasma FRAP value may change or fail to change because of changes occurring in the absolute and relative amounts of UA and/or other antioxidant(s). It would be useful to eliminate the contribution of UA from the samples [28]. In fact, sample type, collection, processing, and methodological limitation must be taken into account when measuring the non-enzymatic antioxidant capacity. Further studies should be performed on individuals with higher BMI and with visceral obesity who are characterized by lower plasma, not salivary, Nu-TAC parameters (Nu-FRAP, Nu-DPPH), particularly examining plasma Nu-TAC indices related to the abdominal distribution of adipose tissue.

It should also be noted that, while both DPPH and FRAP tests measure the TAC, they reflect somewhat different physiological properties. The term TAC is not optimal, since the assay measures only part of the antioxidant capacity and usually excludes enzymatic activities [29]. As neither of the methods for TAC assessment measures all the antioxidants present in body fluids, the simultaneous use of both the FRAP and DPPH assays, as well as their non-urate analogues, enhances the completeness and reliability of the measurements, in spite of their limitations.

The present study has a number of strengths, the key advantage being its simultaneous use of two analytical methods to examine two body fluids, with and without UA, and its use of a number of assessed parameters in groups comparable in age, sex, and anthropometric characteristics; however, it also has some limitations, two of which being the limited number of subjects and the cross-sectional design of the study. It should also be noticed that our subjects were volunteers, who were probably healthier, fitter, and more interested in their health than a random sample, as well as more willing to participate in such studies. Nonetheless, bearing in mind the percentage of subjects with deficient vitamin E (55%) and vitamin C intake (18%), it may be assumed that, despite their mean vitamin C intake being more than adequate, the groups were not as well-nourished as could be expected. Moreover, the heterogeneity of the pharmacotherapy they were subjected to could interfere with the results. It was not feasible to find older subjects entirely free from common age-related ailments or using similar drugs and treatment regimens: the average senior suffers from three or four coexistent diseases. Nevertheless, the diseases diagnosed in our study group were in a stable phase and pharmacologically controlled. Also, some limitations may be associated with the use of a 24 h dietary recall instead of one based on a longer period. Future studies should assess the dietary habits on the basis of longer recall periods, for instance of three or seven days.

A non-supplemented diet based on habitual dietary intake does not significantly affect plasma or salivary Nu-TAC. The known health benefits of a natural, antioxidant-rich diet may be not related to the UA-independent antioxidant potential of plasma or saliva. The measurement of antioxidant capacity in both body fluids, plasma and saliva, with and without UA, provides more information about how the concept of antioxidant capacity could be applied. Further prospective studies are needed to examine these potential relationships.

## 4. Materials and Methods

### 4.1. Patients

The subjects had been treated in the Outpatient Geriatric Clinic of the Medical University of Lodz (Łódź, Poland) and selected from a group of subjects participating in healthy lifestyle workshops organized under the governmental program for the Social Activity of the Elderly (2014–2020) who volunteered to undergo a detailed dietary and laboratory (blood plasma and saliva) assessment. Of the group of 239 community-dwelling seniors participating in the healthy lifestyle workshops, a group of 83 met the inclusion criteria (self-dependent, non-smoking, without active inflammatory process (plasma CRP < 3 mg∙L^−1^), no renal dysfunction, neoplastic disease, disability, or dementia, not using any dietary regimes); these were included in the study group and invited to enter the laboratory stage of the research project. The subjects were consecutively recruited on the basis of inclusion criteria and of the combined value of vitamin C, E, and β-carotene intake (see below) in order to obtain a balanced sex composition of the two groups, differing in the combined intake value of antioxidant vitamins. Because of the failure by some subjects to provide the required volume of saliva/plasma in the sample or the presence of precipitates in the sample, complete data concerning different antioxidant (native and non-urate) parameters were only received from 56 patients (66.9 ± 4.3 years), 82% of whom were females.

Some patients suffered from hypercholesterolemia (*n* = 30), arterial hypertension (*n* = 28), osteoarthritis (*n* = 25), thyroid insufficiency (*n* = 18), osteoporosis (*n* = 14), diabetes mellitus (*n* = 12), duodenal and gastric conditions (*n* = 11), and heart failure (*n* = 7). All diagnosed diseases were in stable phase and pharmacologically controlled. The treatment usually involved statins (*n* = 18), levothyroxine (*n* = 18), angiotensin-converting enzyme inhibitors (*n* = 17), diuretics (*n* = 15), β-blockers (*n* = 12), aspirin (*n* = 10), calcium channel blockers (*n* = 7), proton pump inhibitors (*n* = 6), oral antidiabetic drugs—metformin (*n* = 7), and sulfonylureas (*n* = 4).

The study had been approved by the local ethics committee (RNN/73/15/KE), and informed consent was obtained from each subject. The investigations were carried out following the rules of the Declaration of Helsinki of 1975, revised in 2008.

### 4.2. Study Protocol and Measurements

The examinations took place in the Department of Geriatrics, and the laboratory measurements were performed in the Department of Clinical Physiology, in the Central Scientific Laboratory, and in the University Hospital and Educational Center, at the Medical University of Lodz. The subjects reported to the Center between 8.00–10.00 a.m. after overnight fasting and rest for at least 12 h before blood and saliva collection. The time window between teeth cleaning and non-stimulated saliva sample collection was never shorter than 1.5 h. A comprehensive assessment, including age, sex, drug use, smoking, and dietary habits was performed in each subject [30]. A 24 h dietary recall from the day before the examination was obtained from each individual.

#### 4.2.1. Anthropometric Data

Height and weight were measured, and the Body Mass Index (BMI) was calculated (overweight was for BMI in the range 25–30 kg∙m^−2^, obesity for BMI over 30 kg∙m^−2^). Measurements of waist and hip circumference were taken, and Waist-to-Hip Ratio (WHR) was computed as an index of visceral obesity (diagnosed if WHR > 0.8 in females or >1.0 in males).

#### 4.2.2. Plasma UA, CRP, and Lipid Profile Determinations

Blood samples (approximately 9 mL) were drawn from the antecubital vein and collected for further TAC measurements into Vacuette tubes with lithium heparin or into vacutainer tubes with K3 EDTA for other tests (Vacutest Kima, Arzergrande, Italy). Thereafter, the samples were incubated for 30 min at 37 °C and then centrifuged (10 min, 4 °C, 2880× *g*). The resultant plasma samples for TAC and Nu-TAC measurements (about 4 mL) were stored at −80 °C for no longer than three months [31,32], and the rest was used to assess UA, CRP concentration, and lipid profile parameters.

Enzymatic methods were used to determine plasma total cholesterol (TCh), TG, and UA concentration (BioMaxima S.A. diagnostic kit, Lublin, Poland with Dirui CS-400 equipment). HDL-Ch was measured by the precipitation method (BioMaxima S.A. diagnostic kit). LDL–Ch was estimated using the Friedewald formula. Plasma CRP was measured by an immunoassay (BioMaxima S.A. diagnostic kit, Lublin, Poland with Dirui CS-400 analyzer, Jilin, China).

#### 4.2.3. Plasma TAC and Nu-TAC

Plasma TAC measurements were performed using two spectrophotometric methods: Ferric Reducing Ability of Plasma (FRAP) [33] with some modifications [31] and 2.2-diphenyl-1-picryl-hydrazyl test (DPPH) [31,32]. The coefficient of variation was 4.2% for DPPH. Intra- and inter-assay coefficients of variation for FRAP were less than 8%. Both tests were executed with native (containing UA) and with non-urate plasma samples (Nu-FRAP, Nu-DPPH), i.e., pretreated with uricase and catalase (with decomposed UA). The details of all methods are described elsewhere [12,15,31,34].

#### 4.2.4. Salivary TAC and Nu-TAC

Salivary TAC was also measured spectrophotometrically, using the same equipment (Ultrospec III with Spectro-Kinetics software—LKB Biochrom Pharmacia, Cambridge, UK). The methods used were the same as for native plasma TAC, i.e., Ferric Reducing Ability of Saliva (FRAS) and 2.2-diphenyl-1-picryl-hydrazyl test of saliva (DPPHS), and as for non-urate plasma TAC, i.e., Nu-FRAS and Nu-DPPHS. The details are presented elsewhere [12,16].

To enhance the data reliability, all results were calculated as a mean from three separate experiments. The salivary and plasma native as well as non-urate TAC assays were performed within the same time frame.

#### 4.2.5. Salivary UA and CRP

Salivary UA (SUA) was analyzed using the MaxDiscoveryTM Uric Acid Assay Kit (Bioo Scientific, Austin, TX, USA) based on the colorimetric reaction with a chromogenic dye using peroxidase to form a visibly colored (red) dye product. The result of the absorbance, measured at λ = 520 nm, was proportional to SUA concentration [35].

The salivary CRP assays (ELISA Kit—Salimetrics, State College, PA, USA) were also based on the colorimetric CRP peroxidase reaction on the substrate tetramethylbenzidine. The optical density was read at λ = 450 nm. The amount of CRP peroxidase detected was directly proportional to the amount of CRP present in the saliva sample [36].

#### 4.2.6. Plasma and Salivary ∆ TAC

The percentage of UA/SUA contribution to plasma (∆ FRAP, ∆ DPPH) and salivary (∆ FRAS, ∆ DPPHS) TAC was a measure of the change in TAC after excluding UA or SUA (∆ TAC was the result of the difference between the value of native TAC and the value of Nu-TAC divided by the value of native TAC, expressed in %).

#### 4.2.7. Nutritional Evaluation

A 24 h recall questionnaire was used to register and then encode the intake of food, beverages, and supplements during the preceding day. The intake of energy, nutrients, vitamins, and minerals in the DFR was calculated using the Diet 5.0 software package (developed by the National Food and Nutrition Institute, Warsaw, Poland) and compared with recommendations [37,38]. The degree of insufficient intake of the analyzed antioxidative vitamins was estimated according to the following age and sex standards: EAR (the Estimated Average Requirement) for vitamin C (<60 mg/<75 mg, for females/males respectively) and AI (the Adequate Intake) for vitamin E (<8 mg/<10 mg) [37]. No dietary advice was given before a 24 h recall.

A further extra comparative analysis was run between the two subgroups. Based on a median (Me) value of vitamin C, E, and β-carotene intake, a patient received ‘0’ (if the intake was < Me) or ‘1’ point (if the intake was ≥ Me). Next, the points were added, and, on the basis of the sum result (min = 0, max = 3), the group was divided into group 1 (*n* = 28), with lower vitamin intake (∑ = 0 or 1), and group 2 (*n* = 28), with higher vitamin intake (∑ = 2 or 3). The two numerically matched subgroups were also identical with regard to their sex profile (18% of males in each subgroup).

### 4.3. Statistical Analysis

A power calculation was performed to estimate the required sample size on the basis of data from a prior study [16]. It was found that the sample size required to detect either previously observed differences between groups or minimum differences of 20% with regard to FRAP, DPPH, FRAS, and DPPHS (with a power of 0.80, 0.05 α, type I error rate, and given prior mean and standard deviation values) was 12, 20, 25, and 28, respectively. The data were verified for normality of distribution and equality of variances. The variables that did not meet the assumption of normality were analyzed with non-parametric statistics. Correlations between nutrient intake and age, BMI, WHR, lipid, and antioxidant (native and non-urate) indices in plasma, and antioxidant (native and non-urate) indices and CRP in saliva, were analyzed with the Spearman’s rank correlation coefficient. The Mann–Whitney test was used to compare the mean values of numerical variables between group 1 and group 2. The results of the quantitative variables were presented as a mean ± standard deviation (SD), and *p* < 0.05 was considered statistically significant for all analyses. The statistical analysis was performed using Statistica version 10 CSS software (StatSoft Polska Sp. z o.o., Kraków, Poland).

## Figures and Tables

**Table 1:** Baseline characteristics of the study groups.

Variable	Group 1 (*n* = 28)	Group 2 (*n* = 28)	*p*
Age (years)	67.2 ± 4.4	66.5 ± 4.3	NS
BMI (kg∙m^−2^)	29.9 ± 5.4	28.8 ± 5.1	NS
Waist circumference (cm)	94.9 ± 14.5	90.8 ± 12.7	NS
WHR	0.88 ± 0.09	0.87 ± 0.10	NS
TCh (mg dL^−1^)	175.6 ± 38.7	189.8 ± 34.8	NS
LDL–Ch (mg dL^−1^)	108.0 ± 33.1	124.9 ± 31.1	<0.05
HDL–Ch (mg dL^−1^)	44.2 ± 14.5	46.6 ± 13.6	NS
TG (mg dL^−1^)	132.3 ± 46.3	102.1 ± 55.8	<0.05

Data are presented as mean ± SD. BMI—Body Mass Index; WHR—Waist-to-Hip Ratio; TCh—Total Cholesterol; LDL-Ch—Low-Density Lipoprotein Cholesterol; HDL-Ch—High-Density Lipoprotein Cholesterol; TG—Triglycerides; NS—non-significant difference.

**Table 2:** Plasma and salivary non-urate and native antioxidant indices for group 1 (with lower combined vitamins C, E, and β-carotene intake) and group 2 (with higher combined vitamins C, E, and β-carotene intake).

Variable	Group 1 (*n* = 28)	Group 2 (*n* = 28)	*p*
FRAP (mmol FeCl_2_ L^−1^)	1.25 ± 0.23	1.15 ± 0.16	NS
Nu-FRAP (mmol FeCl_2_ L^−1^)	0.43 ± 0.09	0.40 ± 0.05	NS
DPPH (% reduction)	25.2 ± 5.7	22.1 ± 4.7	<0.05
Nu-DPPH (% reduction)	5.4 ± 1.5	5.7 ± 2.4	NS
FRAS (mmol FeCl_2_ L^−1^)	6.81 ± 2.85	5.22 ± 2.11	<0.05
Nu-FRAS (mmol FeCl_2_ L^−1^)	1.58 ± 1.01	1.42 ± 0.69	NS
DPPHS (% reduction)	27.6 ± 12.6	24.6 ± 12.8	NS
Nu-DPPHS (% reduction)	5.7 ± 4.1	6.5 ± 5.2	NS

Data are presented as mean ± SD. Nu-FRAS—Non-urate Ferric Reducing Ability of Saliva; FRAS—Ferric Reducing Ability of Saliva; Nu-FRAP—Non-urate Ferric Reducing Ability of Plasma; FRAP—Ferric Reducing Ability of Plasma; Nu-DPPHS—Non-urate 2.2-diphenyl-1-picryl-hydrazyl test of saliva; DPPHS—2.2-diphenyl-1-picryl-hydrazyl test of saliva; Nu-DPPH—Non-urate 2.2-diphenyl-1-picryl-hydrazyl test of plasma; DPPH—2.2-diphenyl-1-picryl-hydrazyl test of plasma; NS—non-significant difference.
